# Evaluation of biochemical, hematological and oxidative parameters in mice exposed to the herbicide glyphosate-Roundup^®^

**DOI:** 10.2478/v10102-012-0022-5

**Published:** 2012-09

**Authors:** Raquel Jasper, Gabriel Olivo Locatelli, Celso Pilati, Claudriana Locatelli

**Affiliations:** 1 Curso de Farmácia, Universidade Alto Vale do Rio do Peixe, Rua Victor Baptista Adami 800, Centro, Caçador, SC, Brazil; 2 Universidade Federal de Pernambuco, Programa de Pós-Graduação em Biotecnologia Industrial, Departamento de Antibióticos, Av. Professor Moraes Rego, Cidade Universitária, Recife, PE, Brazil; 3 Departamento de Medicina Veterinária, Centro Agroveterinário, Universidade do Estado de Santa Catarina, Lages, SC, Brazil; 4 Curso de Farmácia, Universidade do Oeste de Santa Catarina, Rua Paese 198, Bairro Universitário, Videira, SC, Brazil

**Keywords:** Glyphosate, Roundup^®^, hepatotoxicity, hematological damage, oxidative stress

## Abstract

We evaluated the toxicity of hepatic, hematological, and oxidative effects of glyphosate-Roundup^®^ on male and female albino Swiss mice. The animals were treated orally with either 50 or 500 mg/kg body weight of the herbicide, on a daily basis for a period of 15 days. Distilled water was used as control treatment. Samples of blood and hepatic tissue were collected at the end of the treatment. Hepatotoxicity was monitored by quantitative analysis of the serum enzymes ALT, AST, and γ-GT and renal toxicity by urea and creatinine. We also investigated liver tissues histopathologically. Alterations of hematological parameters were monitored by RBC, WBC, hemoglobin, hematocrit, MCV, MCH, and MCHC. TBARS (thiobarbituric acid reactive substances) and NPSH (non-protein thiols) were analyzed in the liver to assess oxidative damage. Significant increases in the levels of hepatic enzymes (ALT, AST, and γ-GT) were observed for both herbicide treatments, but no considerable differences were found by histological analysis. The hematological parameters showed significant alterations (500 mg/kg body weight) with reductions of RBC, hematocrit, and hemoglobin, together with a significant increase of MCV, in both sexes of mice. In males, there was an important increase in lipid peroxidation at both dosage levels, together with an NPSH decrease in the hepatic tissue, whereas in females significant changes in these parameters were observed only at the higher dose rate. The results of this study indicate that glyphosate-Roundup^®^ can promote hematological and hepatic alterations, even at subacute exposure, which could be related to the induction of reactive oxygen species.

## Introduction

Glyphosate (N-phosphonomethyl-glycine) is a post-emergence herbicide used for weed control in various crops, especially rice, maize and soybean (Smith & Oehme, 1992; Coutinho *et al.*, 2005). Numerous commercial formulations containing glyphosate as the active ingredient have become popular worldwide, due to high effectiveness and relatively low toxicity to mammals (Corbera *et al.*, 2005). Roundup^®^, one of the most widely used products containing glyphosate, is classified as hazardous to the environment. It was launched onto the American market in 1998 for the control of weeds in sugar cane, coffee, and citrus plantations. In comparison with other formulations, the main characteristic of this product is its rapid absorption, aided by the presence of surfactants. Roundup^®^ contains a mixture of 15% polyoxyethylene amine (POEA) with other unspecified surfactants (Howe *et al.*, 2004). Previous studies have reported that this formulation is more toxic than glyphosate alone (Williams *et al.*, 2000; Howe *et al.*, 2004; Santos *et al.*, 2005). Nonetheless, the literature remains sparse concerning the toxicity of Roundup^®^, especially towards mammals.

Regulatory agencies and scientific institutions worldwide have concluded that glyphosate does not present a risk to human health (Williams *et al.*, 2000). However, recent studies have suggested that long-term exposure to the chemical can cause toxicity in pregnant rats, with bone development deficiency in the fetus (Dallegrave, 2003), changes in cellular metabolism (Marc *et al.*, 2004a; 2004b), cutaneous lesions (Amerio *et al.*, 2004), and increased rates of non-Hodgkin's lymphoma (De Ross *et al.*, 2003). Furthermore, studies using low doses of glyphosate-Biocarb^®^ have shown that the product can cause significant hepatic changes, as well as nasal bleeding without interfering in platelet aggregation (Benedetti *et al.*, 2004; Neiva *et al.*, 2010).

Hematological parameters, such as hematocrit, hemoglobin, and numbers of erythrocytes and white blood cells, can be used as indicators of toxicity and have a broad potential application in environmental and occupational monitoring (Sancho *et al.*, 2000; Barcellos *et al.*, 2003). Biochemical markers of hepatic and renal function, as well as of oxidative stress, are important for biomonitoring the exposure to environmental pollutants (Ahmad *et al.*, 2004).

Many pollutants can induce damage in biological systems, including the mammalian liver, which is the main site in the body for detoxification and biotransformation processes. These involve formation of reactive oxygen species (ROS) such as hydrogen peroxide (H_2_O_2_), the superoxide anion (O_2_^–^), and the hydroxyl radical (**·**OH) (Ahmad *et al.*, 2000; Harish & Murugan, 2011). Due to their high reactivity, these species can damage lipids, proteins, carbohydrates, and nucleic acids (Avellar *et al.*, 2004), leading to serious damage to health.

In order to neutralize ROS, animals possess an antioxidant defense mechanism composed of enzymes including superoxide dismutase (SOD), catalase (CAT), glutathione peroxidase (GPx), and glutathione reductase (GR), as well as non-enzymatic antioxidants including non-protein thiols, especially glutathione (GSH). When the defenses of the organism are insufficient for neutralizing the ROS, oxidative damage can occur, one of the most serious types of which is membrane lipid peroxidation (Scandalios, 2005). This has been reported in several species of fish (Sevgiler *et al.*, 2004; Glusczak *et al.*, 2006; 2007; Modesto & Martinez, 2010a). Meanwhile, the activities of antioxidant enzymes, as well as the occurrence of oxidative damage, have been proposed as indicators of oxidative stress caused by pollutants (Ahmad *et al.*, 2000; Li *et al.*, 2003).

Given the increasing use of glyphosate-Roundup^®^, along with the lack of information on its toxicity in mammals, the objective of this work was to evaluate the effects of the product on hematological, biochemical, and oxidative stress parameters, using male and female albino Swiss mice.

## Materials and methods

### Chemicals

The animals were treated using the commercial glyphosate formulation Roundup Original^®^ (Monsanto, St, Louis, MO, USA), which contains 41% glyphosate as the active ingredient, and 16% polyethoxylene amine as surfactant. The compounds 5,5’-dithiobis(2-nitrobenzoic) acid, reduced glutathione (GSH), malondialdehyde, and thiobarbituric acid (TBA) were obtained from Sigma (St. Louis, MO, USA). All other chemicals used were of the highest grade available commercially.

### Animals

Adult male and female albino Swiss mice, aged 90 days and weighing around 25 g, were housed in plastic cages containing a layer of sawdust that was changed every 3 days to maintain hygienic conditions. Throughout the experimental period, the animals were kept in colonies, with free access to water and food. The temperature was controlled at 22±2 °C, and an illumination cycle of alternating 12-hour periods of light and dark was used.

### Treatment

The animals were organized into three groups of 10 individuals each (both sexes). The control group received distilled water, while the test groups received either 50 or 500 mg/kg body weight of Roundup^®^ diluted in distilled water. The herbicide was administered orally, by gavage, on a daily basis for a period of 15 days. Collections of blood and hepatic tissue were made at the end of the period. All animal experiments were conducted in accordance with the guidelines published by the Society of Toxicology in July 1989 (Guiding Principles in the Use of Animals in Toxicology), and all experiments were approved by the Committee for the Ethical Use of Animals, Universidade Alto Vale do Rio do Peixe (UNIARP, protocol 010/2010).

### Biochemical evaluation

The blood was first centrifuged at 1,500 × *g* for 10 min at ambient temperature. The serum was then separated and used for liver function assessment employing measurements of the enzymes aspartate aminotransferase (AST), alanine aminotransferase (ALT), and gamma-glutamyl transferase (γ-GT). Renal function was evaluated using serum concentrations of urea and creatinine. These tests were performed using disposable kits obtained from Labtest Diagnóstica S.A. (Lagoa Santa, Minas Gerais, Brazil).

### Histopathological analysis

Samples of hepatic tissue were obtained from the animals by surgical excision following euthanasia. In all cases, a standardized 0.5 cm section of sample was removed from the same hepatic lobe. The samples were fixed using 0.1 M phosphate buffer solution (pH 7.4) containing 10% formaldehyde, then washed, dehydrated in alcohol, clarified using xylene, and mounted in paraffin blocks. The tissues were sectioned into 5 µm slices, stained with hematoxylin-eosin, and evaluated by electron microscopy.

### Indices of oxidative stress

Lipoperoxidation in the hepatic tissue was evaluated using the thiobarbituric acid reactive substances (TBARS) technique described by Bird and Draper (1984) in which malondialdehyde and the final products of lipid peroxidation react with barbituric acid, forming a colored complex. The tissue samples were homogenized in 10 mM phosphate buffer (pH 7.0, 1:10 w/v), containing 150 mM NaCl and 0.1% Triton X-100, using a Potter-Elvehjem glass homogenizer. The mixtures were cooled, and then centrifuged at 10,000 × *g* for 10 min at 4 °C. The supernatant was removed and incubated at 100 °C for 1 h with equal volumes of buffer (60 mM Tris-HCl at pH 7.4, containing 0.1 mM DPTA), 12% trichloroacetic acid and 0.73% thiobarbituric acid. The mixture was cooled and then centrifuged at 10,000 × *g* for 5 min. The absorbance of the supernatant was measured at 535 nm. The concentration of TBARS in the sample was calculated from the malondialdehyde analytical curve and the results were expressed as nM/g of tissue.

The concentration of non-protein thiols (NPSH) was determined as described by Ellman (1959). This method is based on the reaction of NPSH with 5,5^’^-dithiobis(2-nitrobenzoic acid) (DTNB), generating the thiolate anion (TNB), which can be measured spectrophotometrically at 412 nm. Samples of hepatic tissue were homogenized in 12% trichloroacetic acid (1:10, w/v), using the Potter-Elvehjem homogenizer. The samples were then centrifuged at 10,000 × g, 4 °C for 10 min and the supernatant was added to the reaction medium (20 µM of DTNB, and 200 mM of sodium phosphate buffer, at pH 8.0). After 10 min at ambient temperature, the absorbance was measured at 412 nm. The concentration of NPSH was calculated using the GSH analytical curve and the results were expressed as mM/g of tissue.

### Hematological evaluation

Hematological parameters, such as red blood cell (RBC), white blood cell (WBC), lymphocyte and neutrophil counts were determined according to Garg and Goyal (1992). The serum content of hemoglobin, hematocrit, mean corpuscular hemoglobin (MCH), mean corpuscular volume (MCV) and mean corpuscular hemoglobin concentration (MCHC) were determined according to Pari and Murugavel (2005).

### Statistical analysis

The results were expressed as mean ± standard error of the mean. Differences between the groups were determined using one-way ANOVA, followed by Duncan's test where appropriate. Significant differences were indicated by *p*-values ≤ 0.05.

## Results

**Figure 1:**
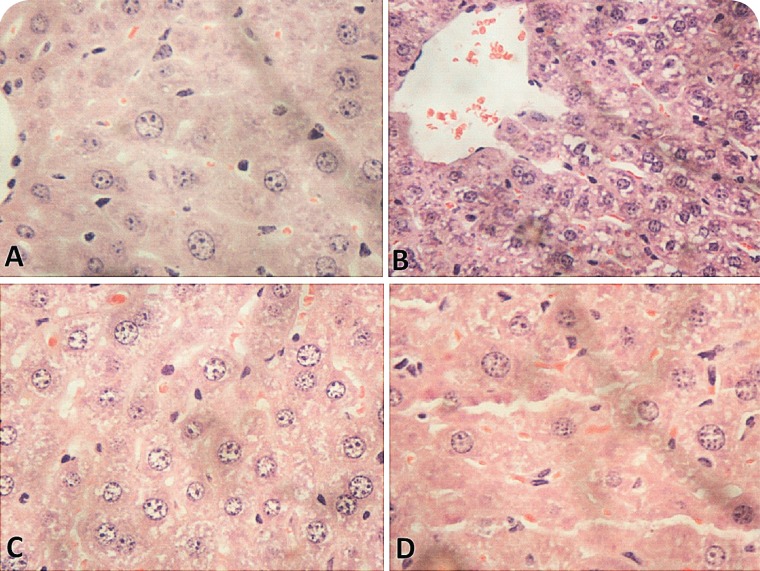
Histological analysis (at ×400 magnification) of the liver lobes of male and female mice submitted to oral treatment with Roundup^®^ for 15 days at a dose rate of 500 mg/kg body weight. (**A**) Female control, (**B**) male control, (**C**) treated female, (**D**) treated male.

The results showed that glyphosate-Roundup^®^ can affect hepatic metabolism, causing important hematological alterations and oxidative damage to the hepatic tissue. Assessment of hepatic and renal biochemical parameters showed that at both concentration levels employed, the glyphosate-Roundup^®^ formulation induced significant liver damage, as indicated by increased levels of the enzymes ALT, AST, and γ-GT, in both male and female mice (Table 1). Nonetheless, histological analysis of the hepatic tissue did not reveal any significant differences compared to the control samples (Figure 1).

**Table 1:** Biochemical parameters of male and female mice submitted to treatment with Roundup^®^ for 15 days.

	MALE			FEMALE		
Parameters	Control	50 mg/kg	500 mg/kg	Control	50 mg/kg	500 mg/kg
**ALT** (IU/L)	67±3.7	79±5*	89±4.3*	41±6.7	58±4*	66.2±4.5*
**AST** (IU/L)	89±9.8	110±8*	130±3.8*	51±4.8	68±5*	93±5.8*
**γ-GT** (IU/L)	634±37	698±27*	734±36*	530±35	638±28*	680±38*
**Urea** (mg/dL)	63±4.3	68±8	69±7.1	60±8.3	61±7	59±2.6
**Creatinine** (mg/dL)	0.45±0.02	0.47±0.04	0.44±0.017	0.46±0.02	0.48±0.04	0.51±0.017

* Significant difference relative to the control (*p≤*0.05).

The liver damage could be related to the capacity of glyphosate to cause oxidative stress since it induced lipoperoxidation and reduced the levels of non-protein thiols in the hepatic tissue (Table 2).

**Table 2:** Oxidative stress in hepatic tissues of male and female mice submitted to treatment with Roundup^®^ for 15 days.

	MALE			FEMALE		
Parameters	Control	50 mg/kg	500 mg/kg	Control	50 mg/kg	500 mg/kg
**TBARS** (nM/g tissue)	94±18	180±15*	303±57*	184±17	230±19*	404±29*
**NPSH** (mM/g tissue)	15±2	12±0.7*	11±0.88*	12.7±0.5	11±1.2	10.3±0.84*

* Significant difference relative to the control (*p≤*0.05).

**Figure 2:**
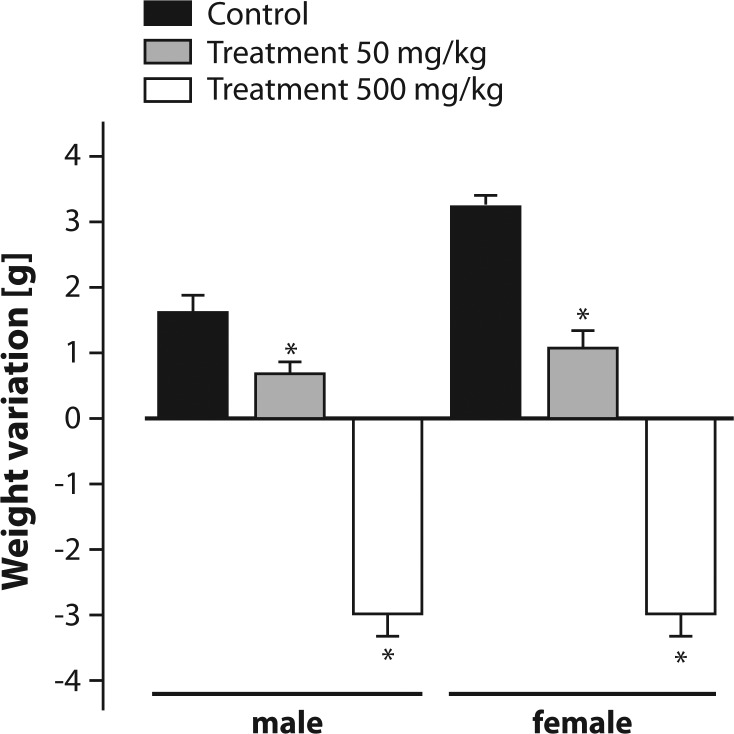
Weight gains of male and female mice submitted to oral treatment with Roundup^®^ for 15 days. Results are expressed as mean ± SEM (n=10). * Significant difference relative to the control (*p≤*0.05).

The animals treated with Roundup^®^ at a dose of 50 mg/kg body weight showed lower weight gain over the 15-day experimental period compared to the controls (Figure 2). Over the same period, the animals that received 500 mg/kg body weight showed significant weight reduction of ∼10% (Figure 2).

Table 3 summarizes the blood parameters of all groups. Our result data show that the median values of blood parameters decreased in animals treated with Roundup^®^ at a dose of 500 mg/kg body weight, indicative of anemic syndrome. In both sexes, there was a significant reduction in the number of erythrocytes and of hemoglobin concentration, with reduced hematocrit and increased MCV, characteristic of macrocytic anemia.

**Table 3:** Hematological parameters of male and female mice submitted to treatment with Roundup^®^ for 15 days.

Parameters	MALE			FEMALE		
	Control	50 mg/kg	500 mg/kg	Control	50 mg/kg	500 mg/kg
**Total RBC count** (×10^6^ mm^3^)	5,200±0.43	5,000±0.45	3,330±0.27*	5,500±0.7	4,800±0.35	3,800±0.25*
**WBC** (mm^3^)	8,422±183	8,225±189	7,900±100	3,500±183	4,200±112	4,000±100
**MCV** (fL)	93±2.5	90±3	105±3.6*	88±5.5	92±4.3	102±5.6*
**MCH** (pg)	29±2.8	30±2	36±2	29±2.8	31±2	34±2
**MCHC** (%)	31±2	33±3	34±3	33±2	34±3	34±3
**Hemoglobin** (g/dL)	15±0.3	15±1	12±1.0*	16±0.6	15±1.2	13±0.5*
**Hematocrit** (%)	48.2±1.8	46±1.5	35±2.02*	48.5±0.63	44±2	38.7±1.8*
**Neutrophil** (%)	18±2	20±2	28±4*	14±2	17±2	19±3*
**Mononuclear** (%)	78±3	79±4	70±4	86±7	82±4	81±5
**Eosinophil** (%)	3±0.5	1±1	4±1	1±0.5	2±1	1±1

* Significant difference relative to the control (*p≤*0.05).

RBC: red blood cell number; WBC: leukocytes count; MCV: mean corpuscular volume; MCH: mean corpuscular hemoglobin; MCHC: mean corpuscular hemoglobin concentration.

## Discussion

Previous works have shown that the toxic effects of different formulations based on glyphosate may be associated with liver and oxidative damage. However, few studies were performed using mammals and most of the earlier works used aquatic organisms sensitive to the herbicide (Lushchak *et al.*, 2009; Modesto & Martinez, 2010a; 2010b; Ortiz-Ordoñez *et al.*, 2011). It is therefore important to investigate the effects of glyphosate on mammals to establish relevant toxicity parameters, as well as to identify possible treatments in cases of occupational or accidental poisoning.

The mechanisms of toxicity of glyphosate formulations are complicated. Not only is glyphosate used as five different salts but commercial formulations of it contain surfactants, which vary in nature and concentration. As a result, human poisoning is not caused by the active ingredient alone but by the herbicide's complex and variable mixtures. It is thus difficult to separate the toxicity of glyphosate from that of the formulation as a whole or to determine the contribution of surfactants to overall toxicity. Experimental studies suggest that the toxicity of the surfactant polyoxyethyleneamine (POEA) is greater than the toxicity of glyphosate and commercial formulations alone. There is insufficient evidence to conclude that glyphosate preparations containing POEA are more toxic than those containing alternative surfactants. Although surfactants probably contribute to the acute toxicity of glyphosate formulations, the weight of evidence is against the suggestion that surfactants potentiate the toxicity of glyphosate (Bradberry *et al.,* 2004).

Since the acute toxicity of glyphosate is increased when the substance is combined with POEA, the present study evaluated the oxidative potential of Roundup^®^ as marketed in Brazil.

Benedetti *et al.* (2004) showed that oral exposure of male Wistar rats to glyphosate-Biocarb^®^ for a period of 75 days increased the levels of the enzymes ALT and AST and induced cellular alterations, as shown by an increase of connective tissue and deposition of collagen in liver cells. Similar findings were observed in the present study, when mice of both sexes were treated with glyphosate-Roundup^®^ for 15 days.

**Schema:**
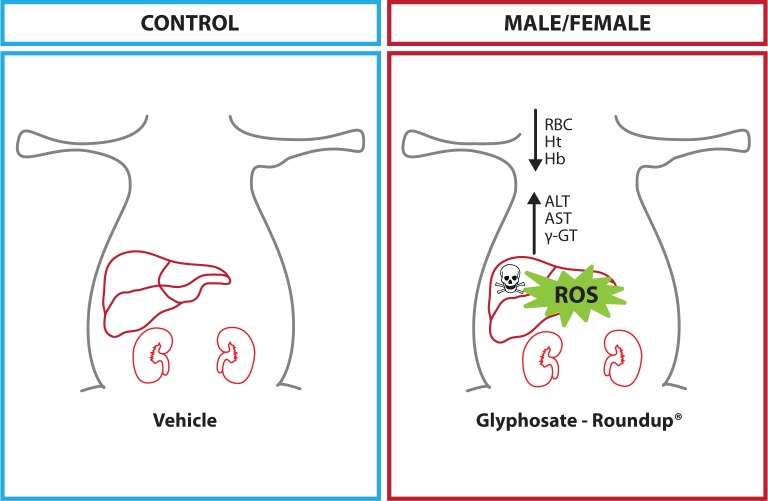
Proposed mechanism of toxicity of glyphosate-Roundup^®^

Cavusoglu *et al.* (2011) administered a single intraperitoneal dose (50 mg/kg body weight) of glyphosate-Roundup^®^ to albino Swiss mice. After 15 days, the mice developed significant hepatic damage, with changes in serum levels of ALT and AST, as well as altered concentrations of urea and creatinine, indicative of hepatic and renal damage. According to the authors, these alterations could have been related to lower levels of glutathione and increased lipoperoxidation.

The liver is the main organ involved in the biotransformation of xenobiotics, and is therefore the site of multiple oxidative reactions, with free radical formation. The liver tissue consequently shows high antioxidant activity, although such activity does not appear to have been sufficient to avoid the damage promoted by glyphosate-Roundup^®^ even at low doses (50 mg/kg body weight). The observed increased lipoperoxidation, together with reductions in the levels of non-protein thiols in hepatic tissue, support the hypothesis that the toxic effect of the herbicide was associated with its capacity to generate ROS. According to Hazarika *et al.* (2003) and Kavitha and Rao (2007), lipoperoxidation can occur through the direct interaction of organophosphorus compounds with the cytoplasmic membrane, resulting in systemic damage. This is the principal molecular mechanism associated with the toxicity of several pesticides.

The association of Roundup^®^ with antioxidants, such as N-acetyl-L-cysteine, vitamins C and E, could reduce toxic damage. A study conducted on cell lines of human keratinocytes (HaCaT) showed that glyphosate and Roundup^®^ were capable of promoting changes in oxidative status. Treatment of cells with the antioxidant vitamins C and E (100 µM) did provide protection from cell death with reduction of IC_50_ and decreasing lipid peroxidation promoted by glyphosate and Roundup^®^ (Gehin *et al.,* 2006).

The impact of antioxidants from dietary combinations can however not be predicted from this *in vitro* investigation. It would therefore be necessary to perform studies *in vivo* with Roundup^®^ plus different antioxidants to confirm either a protective effect or treatment potential against Roundup^®^ intoxication.

Cavusoglu *et al.* (2011) showed that treatment of Swiss albino mice with *Gingko biloba* L. leaf extract (150 mg/kg body weight) produced an improvement in indices of hepatotoxicity, nephrotoxicity, lipid peroxidation, and genotoxicity induced by glyphosate (50 mg/kg body weight). These *in vivo* results showed that *Ginkgo biloba* may present a significant protective effect against the toxicity induced by glyphosate.

Pieniazek *et al.* (2004) demonstrated that glyphosate-Roundup^®^ could increase rates of lipoperoxidation in human erythrocytes. Other authors reported that the herbicide could damage the DNA of different cell lines and the process was apparently associated with increased ROS since there was a higher activity of caspases 3 and 7, which are responsible for the induction of cellular apoptosis (Lioi *et al.*, 1998; Astiz *et al.*, 2009).

Studies using aquatic organisms support the hypothesis that the toxic effects of glyphosate are related to ROS. The herbicide was found to induce increased activity of the antioxidant enzymes GPx, GR, SOD and CAT, as well as glutathione-S-transferase (GST), an enzyme responsible for biotransformation processes, while reducing levels of GSH and increasing lipoperoxidation (Contardo-Jara *et al.*, 2009; Lushchak *et al.*, 2009; Guilherme *et al.*, 2010; Puértolas *et al.*, 2010; Ortiz-Ordoñez *et al.*, 2011).

Lioi *et al.* (1998) identified cytotoxic activity and stimulus of the activity of the glucose-6-phosphate dehydrogenase enzyme in bovine lymphocyte cultures, suggesting that the pesticides tested induced oxidative stress or had mutagenic effects in this species. The hypothesis of oxidative stress has been further reinforced by the findings of Peluso *et al.* (1998), who detected induction of the formation of DNA adducts in the kidney and liver of mice and enhanced hepatic CAT activity in rats treated with glyphosate. Reduced activities of cytochrome P-450 and hepatic monooxygenase were also observed in rats treated with glyphosate-Roundup^®^ (Hietanen *et al.*, 1983).

The production of ROS induced by Roundup^®^ could be the cause of the hematological alterations observed in this study (Table 3). The changes in the hematological parameters suggest that the herbicide caused an anemic syndrome in the animals treated at a dose of 500 mg/kg body weight. Erythrocytes possess limited antioxidant defenses, which renders the cells more sensitive to changes in the antioxidant/pro-oxidant balance. A reduction of GSH and increased lipoperoxidation in these cells can result in cellular lysis.

A significant reduction in the number of erythrocytes observed in mice treated with Roundup^®^ 500 mg/kg body weight may be directly related to the presence of POEA in the formulation. A study showed that active glyphosate had a lower lysis capacity than Roundup^®^ in isolated human erythrocytes (Pieniazek *et al.*, 2004). However no significant lysis in blood from treated mice was observed compared to controls (untreated).

This mechanism is supported by the results of a comparative investigation on pure glyphosate and Roundup^®^. After 1 h of incubation with Roundup^®^, there were increases in methemoglobin, lipoperoxidation, and lysis of human erythrocytes, as well as a higher CAT activity when erythrocytes were treated with 100-1,500 ppm doses of the herbicide. Yet no changes were observed in the level of GSH. It was concluded that Roundup^®^ caused more pronounced changes in erythrocyte function than did the active principle (glyphosate), probably due to the properties of the additives present in the formulation (Pieniazek *et al.*, 2004).

Although *in vitro* studies showed that glyphosate presented some cytotoxicity to human mononuclear peripheral blood cells (Martinez *et al.*, 2007), there were no changes in the total number of leukocytes or in mononuclear cell counts when Roundup^®^ was administered to mice (Table 3). However, we observed a slight increase in the number of leukocytes in female mice associated with a significant increase in neutrophils. This effect observed in females may be related to their higher sensitivity to inflammatory processes.

The more pronounced changes in the number of erythrocytes when compared to leukocytes may be related to the higher sensitivity of erythrocytes to oxidative stress induced by Roundup^®^. Another possibility would be alterations in bone marrow caused by treatment with Roundup^®^. Prasad *et al.* (2009) found that treatment of Wistar rats with glyphosate caused chromosomal aberrations in bone marrow cells after exposure to the herbicide for periods of 24, 48, and 72 h, at doses of 25 and 50 mg/kg body weight. These findings could provide an explanation for the anemia syndrome identified in the present study. Nonetheless, the chromosomal alterations, as well as the reduced numbers of erythrocytes, may have been caused by imbalance between the antioxidant/pro-oxidant defense mechanisms in bone marrow.

Yousef *et al.* (1995) investigated the effect of glyphosate on the semen of rabbits treated with the herbicide and reported declines in body weight, libido, ejaculation volume, spermatozoid concentration, osmolarity and concentration of fructose in the semen. Changes in the body weight were observed also in the present work in mice (Figure 2). According to Chahoud *et al.* (1999), weight loss is an important indicator of toxicity; thus, it can be inferred that Roundup^®^ induced systemic toxicity, which may be associated with its capacity to stimulate ROS production.

Druart *et al.* (2011) showed that exposure of snails to glyphosate at a dosage of 4 mg/kg body weight resulted in reduced growth rates. In another work, Dallegrave *et al.* (2003) found that exposure of pregnant rats (between days 6 and 15 of gestation) to 500 mg/kg body weight of glyphosate reduced fetal growth rate. The authors noted that the reduced growth rate could have been caused by tissue deposition of glyphosate.

Williams *et al.* (2000) reported that in order to achieve significant toxic effects, Sprague-Dawley rats were exposed to Roundup^®^ at doses exceeding 1,000 mg/kg body weight. Such a dose rate greatly exceeds the amounts employed in the present work, in which significant toxic effects were demonstrated. This could be partially explained by the capacity of glyphosate to bioaccumulate, as reported by Contardo-Jara *et al.* (2009).

In summary, the results of the present work indicate that exposure to Roundup^®^, even at low doses and for a relatively short period of time, can induce serious hepatic and hematological damage, caused presumably by increased oxidative stress. The extensive global use of different glyphosate formulations underlines the importance of the findings. Long-term exposure to glyphosate present in contaminated soil or water, even at low concentrations, can lead to serious human health problems, including liver damage, anemia, and conditions associated with ROS, such as different types of cancer and neurodegenerative diseases.
